# Toward general object search in open reality

**DOI:** 10.1038/s41598-025-97251-5

**Published:** 2025-04-19

**Authors:** Gang Shen, Wenjun Ma, Guangyao Chen, Yonghong Tian

**Affiliations:** 1 Smart Tower Co., Ltd., Beijing, China; 2 National Key Laboratory for Multimedia Information Processing, School of Computer Science, Peking University, Beijing, China; 3 School of Electronic and Computer Engineering, Shenzhen Graduate School, Peking University, Beijing, China; 4 Peng Cheng Laboratory, Shenzhen, China

**Keywords:** Mathematics and computing, Computer science

## Abstract

Real-world scenarios are inherently dynamic and open-ended, necessitating that current deep models adapt to general objects in open realities to be practically useful. In this paper, we extend a valuable computer vision task called **G**eneral **O**bject **S**earch in **O**pen Reality (**GOSO**). The main objective of GOSO is to determine whether an object from the open world appears in another gallery image, even when composed of arbitrary entities and backgrounds. However, two significant challenges arise: the high scale variance among different instances of the same entity and the vast openness with an ever-expanding set of unknown categories in the open world. To address these issues, we formalize the GOSO problem and propose a simple yet effective architecture named **S**iamese **E**xchanged **A**ttention Network (SEA-Net). Specifically, based on a standard siamese structure, SEA-Net introduces a novel branch that comprises multiple stage-stacked Siamese Exchanged Attention (SEA) layers followed by a Hierarchical Feature Fusion (HFF) module, enabling efficient scale adaptation and the extraction of matching-friendly deep features. Moreover, an Open Score Fusion (OSF) module is integrated into SEA-Net during inference to yield a more robust matching score in open-world scenarios. We construct two new evaluation benchmarks suitable for the GOSO task using the existing COCO and LVIS datasets, and extensive experiments consistently demonstrate the effectiveness of the proposed method.

## Introduction

**Fig. 1:**
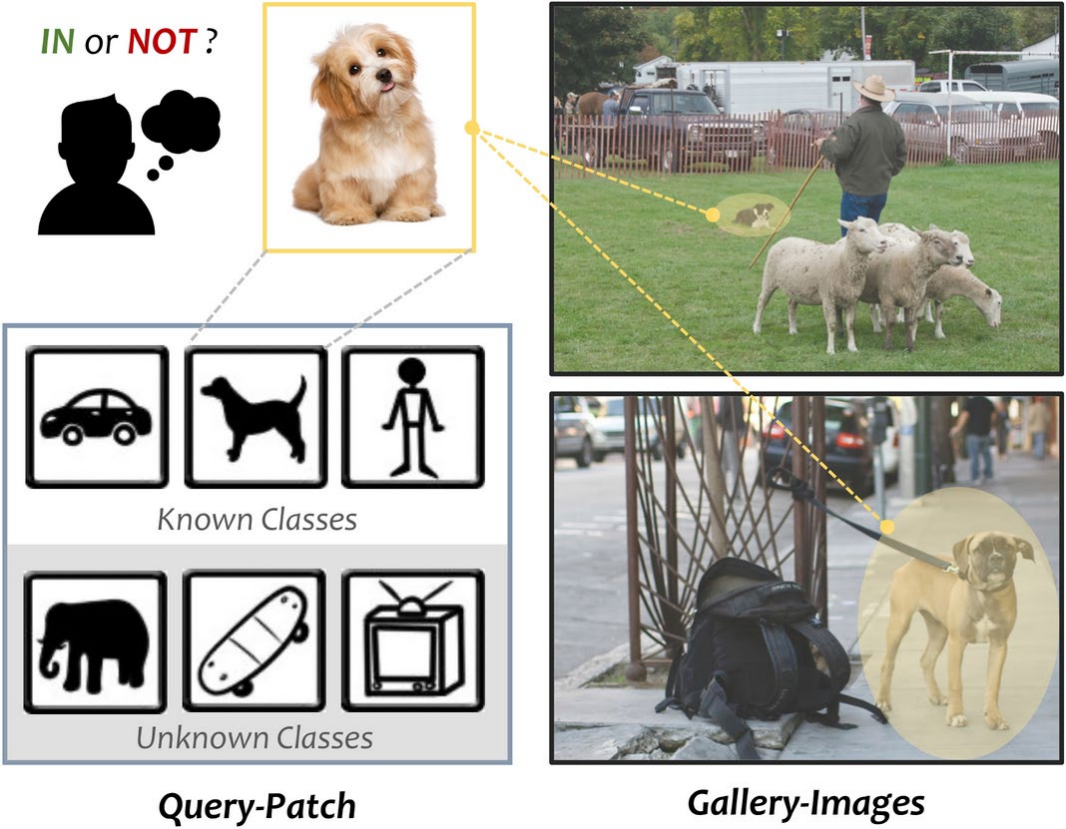
We focus on the problem of whether or not and illustrate the challenges of the General Object Search in Open Reality, i.e., scale variance (yellow area) and entity openness (gray part).

Humans exhibit a powerful ability to search for designated targets in the open world. That is, given a query of *dog*, regardless of whether this class has been seen before or not, even a child can easily determine whether other images contain its objects. This kind of ability is innate to humans, yet it is hard to reach deep networks. Moreover, with the exponential increase in the amount of data, object search is becoming one of the most essential techniques for many downstream vision tasks, such as auto-labeling and so on.

Lately, some related topics have been explored in depth, such as image retrieval and matching^1^, but most of them work under the setting of specific scenes or entities, e.g., pedestrians^2^, vehicle^3^, products^4^ and buildings^1^, which is still far from satisfactory for many real needs. It is of thus motivating us to extend a valuable vision task called General Object Search in Open Reality (GOSO), where a deep model is tasked to *identify whether an object query from the open world appears in another complicated image with arbitrary entities and background or not.* Consider the Fig.1, there are two main challenges in this task: (1) high scale-variance, the object scales of the same category in different gallery images vary greatly, leading to the inability to align between local and global features, and (2) large entity-openness, the category range in the open scenario is progressively expanding, i.e., a coming query may contain objects from unknown space, and it should still be discovered aright even if the model is not updated anymore.

**Fig. 2:**
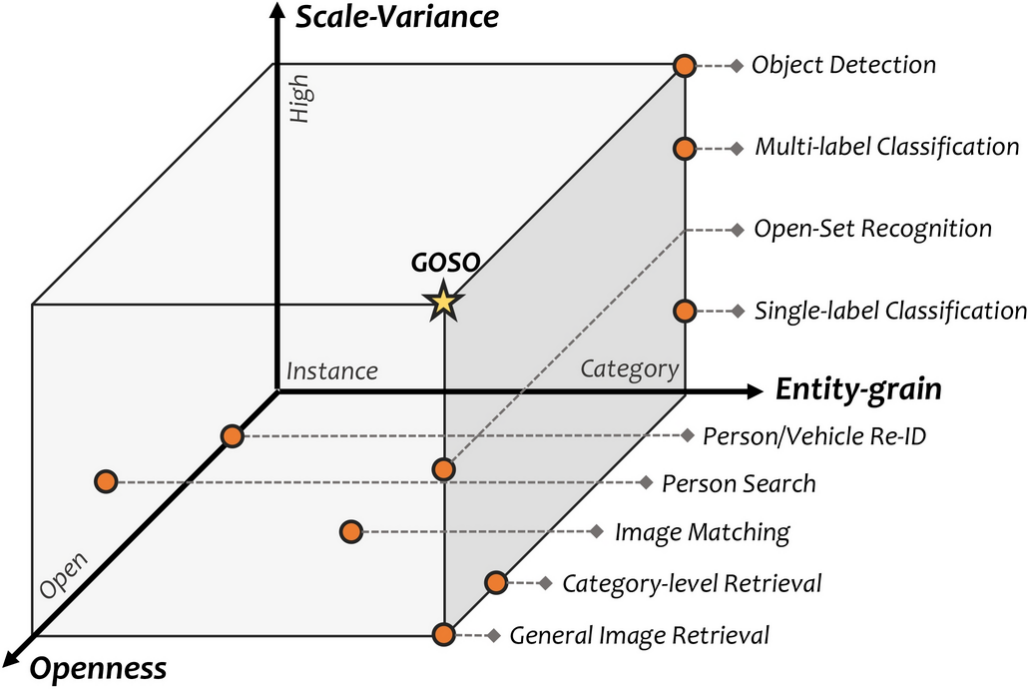
We compared some existing related vision tasks from three perspectives, i.e., scale-variance, openness, and entity-grain. As a valuable topic moving towards open reality, the proposed *GOSO* is still very lacking in exploration now.

Prior to this, the matter of scale-variance has been widely studied in object detection, which often employs the region-of-interest (RoI) pooling to align multi-scale features^5^. Unlike these current detection pipelines with equipping RoI or localization branch, *GOSO* only focuses on the question of *whether or not* without any box priors, which is achieved through feature-level matching with lower complexity. Besides, although the multi-label classification task^6^ can judge which categories are contained in a picture, it only works in the closed setting with seen classes, which greatly limits its application scenarios in open reality. In fact, we argue that openness is one of the criteria for measuring the ability of a task to adapt to open world. In spite of some subtasks of image retrieval, which are formalized as a matching problem, have weak openness in certain specific fields, their paradigms of patch-to-patch (instance-level) or image-to-image (pixel-wise) yet increase the difficulty of directly adapting to general object search. Interestingly, as shown in Fig. 2, due to the *high scale-variance*, *large openness* and *category-level searching*, the *GOSO* is still under-explored.

**Fig. 3:**
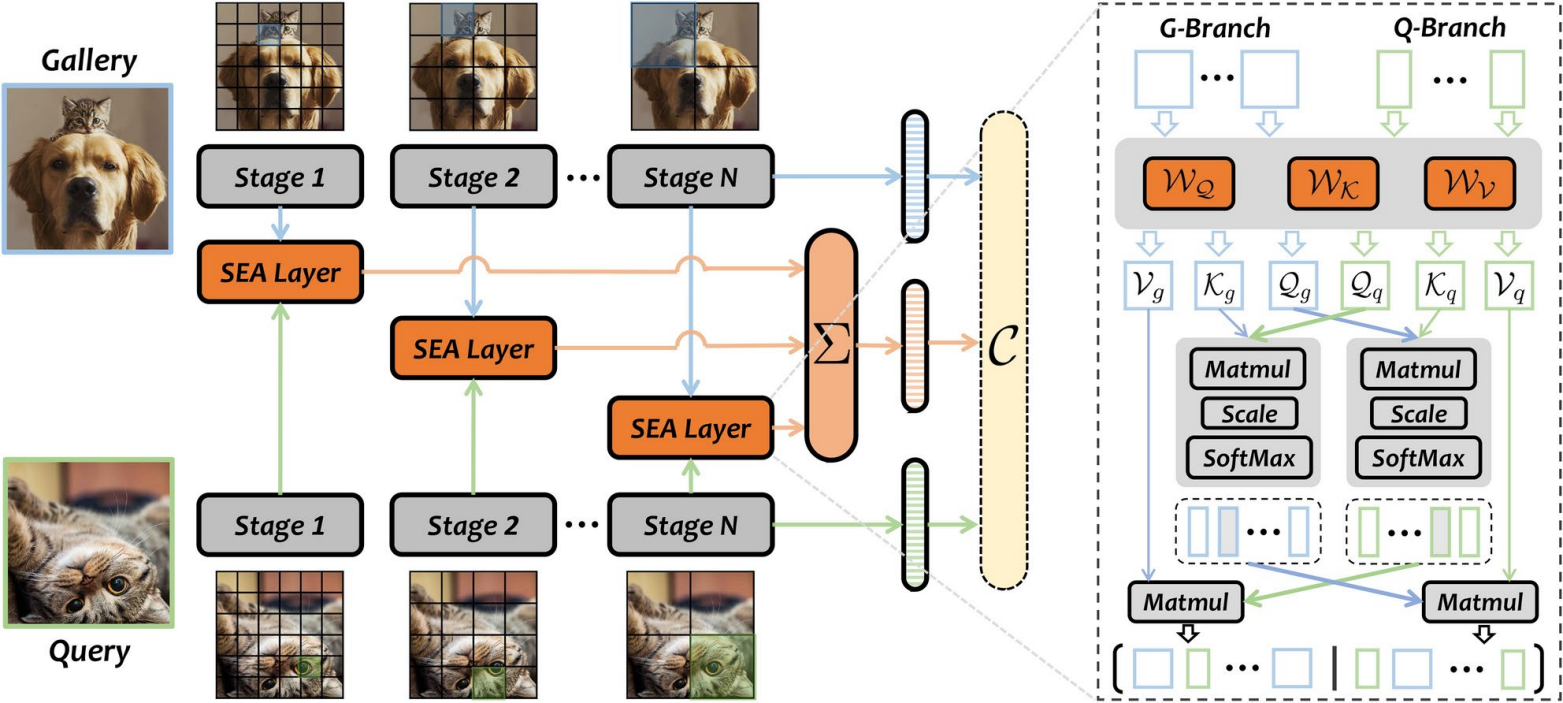
The architecture of Siamese Exchanged Attention Network (*SEA-Net*) for General Object Search in Open Reality (*GOSO*). The model is composed of three parallel branches, two of which share weights and are used to extract the features of gallery and query respectively, termed as *G*-branch (blue) and *Q*-branch (green). The other branch (orange) progressively extracts the desirable features with richer semantic information through multiple stage-stacked *SEA* layers. Then these features are fused by the Hierarchical Feature Fusion module (*HFF*), which is concretized as a weighted summation function ($\Sigma$) in Section “Siamese exchanged attention module”. Note that ${W}_{Q}$, ${W}_{K}$, ${W}_{V}$ are shared by *G*-branch and *Q*-branch in each *SEA* layer. Finally, the features from all three branches are fed into a shared classification layer *C* (yellow) to calculate loss value (during training) or the proposed Open Score Fusion module (*OSF*) in Section “Open score fusion module” to produce matching scores (during inference).

In this work, we propose a simple yet effective approach, named Siamese Exchanged Attention Network (*SEA-Net*), to deal with the essential challenges in *GOSO*. The overall architecture of *SEA-Net* is very straightforward as demonstrated in Fig. 3. Compared with the standard siamese structure, *SEA-Net* additionally adopts another attention branch to perform rich feature interaction and flexible scale adaptation between the query and gallery across multiple stages. From the perspective of intra- and inter-stage, the newly inserted branch consists of two novel modules, i.e., Siamese Exchanged Attention layer (*SEA*) and Hierarchical Feature Fusion module (*HFF*). The former is a symmetric structure that takes the query feature and gallery feature of the other two branches as inputs, see the right part of Fig. 3. Based on the attention mechanism, on the one hand, information interaction is conducted by exchanging query matrices between two inner-branches of *SEA* layer, and on the other hand, the fusion feature with stronger semantics is obtained by performing mutual response on the attention map. The latter one is a weighted summation operator for integrating the output features from multiple stages. Since hierarchical blocks have gradually-scaled receptive fields from low- to high-level, the *HFF* helps to extract features with richer semantics. However, under the setting of *GOSO*, the above branch can only be optimized on known classes, which is far less than unknown data in open reality. In order to narrow the inductive bias on seen spaces, *SEA-Net* further employs an Open Score Fusion module (*OSF*) to produce robust scores for queries from know and unknown spaces.

The main contributions of our approach are four-folds:

- To tackle the matter of *whether or not*, we formally extend a valuable and practical vision task, named *GOSO*, which models the real world more closely.
- We propose a simple yet effective architecture for *GOSO*, called *SEA-Net*, which consists of multiple stage-stacked *SEA* layers, a following *HFF* module and a cost-free *OSF* strategy, to address the challenges of *GOSO* task.
- We curate two large *GOSO* datasets from existing detection datasets, i.e., *COCO* and *LVIS*. We set up benchmarks for comprehensive *GOSO* performance evaluation.
- Extensive experiments on these benchmarks consistently demonstrate the effectiveness of our proposed method.

## Related works

### Content-based image retrieval

Content-based Image Retrieval searches for semantically similar pictures in a large gallery by analyzing their visual content with a query. It is generally divided into two groups:

#### Instance-level image retrieval

Given a query image of a particular object, this task aims at finding images containing the same instance that may be captured under different conditions. With the rise of deep models, most of the recent progresses in this field are based on CNNs. For example, re-identification (ReID) retrieves a query-of-interest across multiple non-overlapping cameras, where the instance can be pedestrian^7–14^ or vehicle^3,15,16^. In addition, clothing retrieval^17^ is also a similar research topic. No matter which one of the above tasks, their query and gallery usually appear in the form of cropped patches, that is, there is no complicated background. However, the gallery image in *GOSO* usually contains more entities and complex scenes, which greatly increases the difficulty of object retrieval. One of the most related to *GOSO* is person search^18–20^, which is usually regarded as an extension of the person ReID with an additional detector in the scenes. Note *GOSO* extends the entity category from pedestrian to arbitrary objects and solves the problem end-to-end.

#### Category-level image retrieval

The goal of this task is to retrieve images including the same category as the query, which focuses more on coarse-grained information than instance-level.^21^ proposes a bidirectional approach to rank for representation learning and^22^ deploys the multiple labels to learn the semantic space. Moreover,^23^ looks into the effectiveness of classification-based approaches on image retrieval tasks and^4^ introduces a cooperative embedding to integrate category-level and instance-level while preserving their specific level of semantic representation. Instead of evaluating on the fine-grained and closed datasets, such as Cars-196^24^, CUB200^25^ and Product^26^, the *GOSO* task shares the same vision with category-level retrieval but focuses more on open scenario.

### Image matching

Image matching is a vision task of identifying then corresponding the similar structure from two images^1^, which generally maintains at the pixel-wise and instance-level correspondence. In particular, the deep feature-based image matching pipeline, which is composed of feature detection, description and then matching sequentially, has been flexibly adopted into 3d-reconstruction^27,28^, VSLAM^29,30^ and image retrieval^31–33^. Due to the image similarity being intrinsically determined by the feature matches between images, the retrieval score can be obtained by aggregating votes from the matched features. Recent advances in re-identification further push the boundaries of image matching. For example,^34^ proposes a novel dual-domain modulation framework that mitigates the domain gap between daytime and nighttime vehicle images by incorporating a glare suppression module, a dual-domain structure enhancement module, and a cross-domain class awareness module. These components help obtain more robust features for cross-domain matching. Similarly,^35^ leverages high-order structural information to learn middle-level features that are effective for matching visible and infrared images. Moreover,^36^ provides a comprehensive review of Transformer-based approaches that, by exploiting both global and local feature correspondences, enhance the robustness of image matching in object re-identification tasks. However, unlike retrieval based on image matching, where the images for matching are taken from the same or similar object while captured at different times or viewpoints, *GOSO* is set to retrieve based on high-level semantics, not pixel-by-pixel.

### Multi-label classification

This task aims to gain a comprehensive understanding of image and then identify all known entities in it accordingly. There are three main research branches: (1) improving the loss function from the perspective of imbalance data^37–39^, (2) mining label correlations from the view of knowledge transfer^40,41^ and (3) digging out more reasonable regions of activation response^42–44^. Due to the closed nature of this topic^6^, all above approaches can only identify known categories, while ignoring meaningful classes of unseen entities. Yet, *GOSO* is essentially a matching problem, that is, to determine whether a picture contains a certain category, including unknown entities.

### Open-world applications

Due to the inductive-bias of deep networks, the performance of most existing approaches on many vision tasks will be severely degraded in open-world scenarios.^45–47^ and^48^ identify unknown classes in classification and detection problems through softmax-variant and contrastive-clustering, respectively. Moreover, some few-shot methods further explore feature adaptation on novel domain^49^ with very few annotated data, such as model-finetuing^50–52^ and feature-attention^53^. As for the category-level retrieval, our *SEA-Net* focus on the multi-scale feature matching, as well as the adaptation on query-shot.

## General object search in open reality

To define the problem of *GOSO* formally, we assume that there are two disjoint datasets ${D}_{K}$ and ${D}_{U}$ in real world, with known classes ${C}_{K} = \{1, \dots , C \}$ and unknown classes ${C}_{U} = \{C+1, \dots \}$ respectively, where *C* is the number of annotated categories and ${C}_{U}$ can only be encountered during inference. Hereafter, let us first consider the training set ${D}_{tr}$ with consisting of a query set ${D}^{Q}_{tr} = \{x_q, y_q\}$ and a gallery set ${D}^{G}_{tr} = \{ x_g, \vec {y}_g\}$, i.e., ${D}_{tr} = \{ {D}^{Q}_{tr}, {D}^{G}_{tr} \}$, where $x_q$ is an object patch (cropped image without background) and $y_q \in {C}_{K}$ is its label, $x_g$ is an image from the open reality and $\vec {y}_g = \{y_i\}_{i=1}^{N}$ is annotated by a label vector, where $y_i \in \{0, 1\}$ denotes whether the class *i* is present in the image (‘1’) or absent (‘0’). As for the testing set ${D}_{te} = \{ {D}^{Q}_{te}, {D}^{G}_{te} \}$, in order to realistically model the dynamics of open world, we further introduce unknown entities into both query set ${D}^{Q}_{te} = \{x_q, y_q\}$ and gallery set ${D}^{G}_{te} = \{ x_g, \vec {y}_g\}$, where $y_q \in {C}_{K} \cup {C}_{U}$ and $\vec {y}_g = \{y_i\}_{i=1}^{N+\mid {C}_{U} \mid }$.

Under this setting, the ultimate goal of our algorithm is to optimize a robust feature extractor *F* based on the known dataset ${D}_{tr}$ and then, given a query $q_{i}$ from ${D}_{te}^{Q}$ (one-shot), the trained model calculates the matching probability $p_{i}$ between the query and a gallery image $g_i$ from ${D}_{te}^{G}$, i.e.,, $p_{i} \leftarrow {F}(q_{i}, g_{i})$. Due to the limited number of seen classes during the training procedure, matching unknown queries correctly requires the model to have strong generalization and adaptability. In addition, in open reality, different objects of the same category, or even the same object while captured at different times or from different viewpoints, are inconsistent in scale. Thus, a scale-insensitive and efficient content-to-content matching module is desirable to establish appropriate correspondences.

As shown in Fig. 2, GOSO is fundamentally a cross-image matching task. Unlike multi-label open-set recognition-which classifies every object within a single image and rejects unknown categories based on global features-GOSO focuses on verifying whether a query object appears in a gallery image using fine-grained content matching.

## Methodology

This section first provides an overview of the *SEA-Net* architecture and then elaborates on the Siamese Exchanged Attention module (*SEA*) to learn a general embedding containing semantic and matching representation, which takes intra- and inter-stage scale adaptation into consideration. Next, the Open Score Fusion (*OSF*) module is proposed to improve the robustness of matching scores in open reality.

### Architecture overview

Figure 3 shows the high-level architectural overview of our method. To be concrete, given a pair of query image ${I}_q$ and gallery image ${I}_g$, a weights-shared backbone is first employed to extract the deep features $x^k_q$ and $x^k_g$ for query and gallery respectively, where *k* represents the *k*-th stage of backbone. Then the features $x^k_q$ and $x^k_g$ from the same stage are fed into the novel Siamese Exchanged Attention layer (*SEA*), which is illustrated in the Fig. 3 (right) in details (see Section “Siamese exchanged attention module”), to perform dense content-to-content matching on feature-level by attention activation. Furthermore, since different stages have gradually-scaled receptive fields, the following Hierarchical Feature Fusion module (*HFF*) summarizes the *SEA* features for richer semantics, $\Sigma$ in Fig. 3, which further alleviates the problem of high scale-variance under the setting of *GOSO*. During training, all these features from three branches are fed into a shared classification layer *C* for predicting labels. With optimizing on the known dataset ${D}^{tr}_{K}$, the overall loss function can be formalized as,

1 $$\begin{aligned} {\begin{matrix} \mathop {\arg \min }_{\theta _c, \theta _{s}}{ {L}({F}({I}_{q}, {I}_{g}; \theta _b, \theta _s, \theta _c), y_q \cap \vec {y}_g)}, \end{matrix}} \end{aligned}$$

where $\theta _b$ , $\theta _s$ and $\theta _{c}$ are the parameters of the siamese backbone, the *SEA* module and the classifier respectively. Note the $\theta _b$ is frozen during finetuning and we choose cross-entropy loss as the supervised function. In inference, the forward pipeline removes the last classifier and then uses the Open Score Fusion module to produce matching scores.

### Siamese exchanged attention module

Attention mechanisms aim to highlight important local regions and extract more discriminative features. Yet, on the one hand, due to learning only on the closed priors of known classes, the conventional attention structure cannot be well generalized to unknown spaces. On the other hand, it is high-complexity to establish a dense region-to-region matching relationship between the separated feature maps from the query branch (*Q*-branch) and gallery branch (*G*-branch), resulting in poor adaptability to the object scale. In this section, we first revisit the existing structure of All-Attention Fusion (*AAF*)^54^ and analyze its shortcomings, and then propose a novel Siamese Exchanged Attention module (*SEA*) with the symmetrical structure to model the semantic relevance between the gallery and query features, ${i.e. },$, $x^k_g$ and $x^k_q$, thus draw scale-insensitive attention to the target objects and benefit the subsequent matching.

#### Revisiting all-attention fusion

As one of the most straightforward approaches for feature interaction, All-Attention Fusion (*AAF*) simply concatenates all features from the query and gallery branches and then integrates the information via a standard self-attention module^55^. This module comprises a multi-head attention mechanism followed by a feed-forward network, with layer normalization (*LN*) applied prior to each block. In our implementation, given an input feature matrix $x_f^k$, the multi-head attention mechanism first projects it into query (*Q*), key (*K*), and value (*V*) matrices using learnable linear transformations. These matrices are then divided into *h* independent heads:

$$Q = [Q_1; \dots ; Q_h], \quad K = [K_1; \dots ; K_h], \quad V = [V_1; \dots ; V_h],$$

where each head operates in a subspace of dimension $d_k = \frac{d}{h}$ (with *d* being the original feature dimension). For each head *i*, the attention output is computed as:

$$\text {head}_i = \text {Attention}(Q_i, K_i, V_i) = \text {softmax}\left( \frac{Q_iK_i^\top }{\sqrt{d_k}}\right) V_i.$$

The outputs from all heads are then concatenated and passed through an additional linear projection to form the final output of the multi-head attention block. In our formulation, this entire multi-head attention process is denoted by ${AAF}^{\dagger }(\cdot )$ in Equation (2). However, due to a lack of branch-specific consideration, this scheme incurs a quadratic computational cost relative to the number of features. The output $z^k$ of *AAF* can be expressed as:

2 $$\begin{aligned} {\begin{matrix} x^k_f & = \left[ x^{k}_{q} \ \Vert \ x^k_{g} \right] , \\ o^k & = x^k_f + {AAF}^{\dagger }({LN}(x^k_f)), \\ z^k & = g(o^k), \end{matrix}} \end{aligned}$$

where $\Vert$ denotes the concatenation operation and $g(\cdot )$ is the back-projection function implemented via an MLP to align the feature dimensions.

#### Siamese exchanged attention layer

In order to integrate the features from *Q*-branch and *G*-branch more efficiently, we propose an extension to attention, named as Siamese Exchange Attention Layer (*SEA*), to exchange information between the *Q*-branch and *G*-branch in a symmetrical manner, which is shown in the right part of Fig. 3. To be concrete, given the representations $x^k_\star$ from the shared feature encoder, where $\star$ can be *q* or *g*, the process starts by adapting inputs for query-key-value attention computation. With utilizing three learnable embedding matrices, ${i.e. },$, ${W}_{Q}$, ${W}_{K}$, ${W}_{V}$, the *SEA* layer first projects the context $x^k_\star$ into the query ${Q}_\star$, the key ${K}_\star$ and the value ${V}_\star$ respectively. Note that the learnable projection matrices are shared between the *Q*-branch and *G*-branch. Hereafter, the interactive attention map is computed via exchanging the query matrix mutually and then performing softmax dot-product between the swapped query ${Q}_g$ (or ${Q}_q$) and the original key ${K}_q$ (or ${K}_g$), which can be viewed as content-to-content interaction between two branches. Finally, *SEA* aggregates the value embeddings ${V}_g$ (or ${V}_q$) with using the corresponding attention map ${A}_q$ (or ${A}_g$) as kernel weights separately and then concatenates the output representations from two branch together as a joint feature for next modules. Mathematically, the *SEA* layer can be formulated as:

3 $$\begin{aligned} {\begin{matrix} & {Q}_q = {x^k_q}{W}_{Q}, \ \ \ {K}_q = x^k_q{W}_{K}, \ \ \ {V}_q = x^k_q{W}_{V}, \\ & {Q}_g = {x^k_g}{W}_{Q}, \ \ \ {K}_g = x^k_g{W}_{K}, \ \ \ {V}_g = x^k_g{W}_{V}, \\ & {A}_q = {softmax}({Q}_g{K}_q^{\top } / \sqrt{C / h} ), \\ & {A}_g = {softmax}({Q}_q{K}_g^{\top } / \sqrt{C / h} ), \\ & SEA(x^k_q,x^k_g) = \left[ {A}_g{V}_q \ \vert \vert \ {A}_q{V}_g \right] , \end{matrix}} \end{aligned}$$

where *C* and *h* are the embedding dimension and the number of heads, ${W}_{Q}$, ${W}_{K}$, ${W}_{V}$ $\in \mathbb {R}^{C\times (C/h)}$ are learnable parameters and shared between *Q*-branch and *G*-branch. Note that typically, since the query matrix *Q* of one branch is replaced by the other branch, the computation and memory complexity of generating the attention map *A* in *SEA* layer are linear rather than quadratic as in *AAF*, which makes the entire process more efficient. Moreover, after further employing the multi-heads mechanism with layer normalization and residual shortcut, the output $z^k$ of *SEA* layer for a given features pair $x^k_q$ and $x^k_g$ is defined as follows:

4 $$\begin{aligned} {\begin{matrix} x^k_f & = \left[ x^{k}_{q} \ \vert \vert \ x^k_{g} \right] \\ o^k & = x^k_f + {SEA}^\dagger ({LN}(x^k_q), \ {LN}(x^k_g)), \\ z^k & = g(o^k), \\ \end{matrix}} \end{aligned}$$

#### Intra- and inter-stage scale adaptation

To alleviate the problem of high scale-variance in *GOSO*, the proposed *SEA* adapts various-scale matching from two orthogonal view, namely Intra- and Inter-stage. As mentioned in the Section “Siamese exchanged attention module”, the feature maps from the same stage of the backbone are fed into the *SEA* module, which can be regarded as conducting dense region-to-region matching between query and gallery with the same receptive field, i.e., intra-stage scale adaptation. Concretely, for an object of any scale in the gallery, part of the content (if exists) on its feature map can always correspond to the query one, and then be activated by attention, which is illustrated by the green shade and blue shade in Fig. 3. Moreover, with the bottom-up feed-forward computation of the backbone, one widely intuition is that deep networks combine low-level features to increasingly complex shapes until the object can be readily discriminated^56^. Therefore, based on the hierarchical structure for different granularity, we apply several *SEA* layers among multiple stages of the model and then fuse them with a Hierarchical Feature Fusion module (*HFF*), that is, inter-stage scale adaptation, which can be formulated as:

5 $$\begin{aligned} {\begin{matrix} {B}^k =\frac{k-1}{N} \cdot {B}^{k-1} + {GAP}({Z}^{k}), k \in [1, N] \\ \end{matrix}} \end{aligned}$$

where *N* is the number of stages in backbone, *GAP* represents the global average pooling function, ${Z}^{k}$ is the output of *k*-th *SEA* layer and ${B}^N$ is the final fusion feature.

### Open score fusion module

Given $x_q$ and $x_g$ under the setting of *GOSO*, with considering the known and unknown spaces simultaneously, a desirable matching score function should be defined as:

6 $$\begin{aligned} {\begin{matrix} {S}_{q \leftrightarrow g} = \phi (f_{{K}}(x_q), f_{{K}}(x_g)) \oplus \phi (f_{{U}}(x_q), f_{{U}}(x_g)) \end{matrix}} \end{aligned}$$

where $f_{K}$ is the convergent parameter space after optimizing on known dataset, $f_{U}$ represents the part of unknown, $\phi$ is a pairwise distance function for similarity score and $\oplus$ defines the final scores fusion operator. According to this definition, we propose the Open Score Fusion module (*OSF*) with concretizing the $f_{K}$, $f_{U}$, $\phi$ and $\oplus$ in Eq. (6).

After finetuning on the known dataset ${D}_{K}^{tr}$, $f_{K}$ actually is the well-parameterized *SEA* module in Section “Siamese exchanged attention module”. In general, due to the unreachability nature of unknown space, we can only approximate the $f_{U}$ for feasibility. The most straightforward way is to omit the $f_{U}$ and then the matching score is completely determined by the $f_{K}$, usually leading to poor performance due to severe overfitting, which has been also empirically verified in the Tables 1 and 2. Another that is not precise but very practical is to use the large-scale pre-trained model (e.g. ImageNet) to close in $f_{U}$, which is cost-free to obtain and friendly for downstream tasks. Furthermore, the pairwise distance function $\phi$ is denoted as cosine similarity and we use summation as the score fusion operator. As stated above, the overall *OSF* module can be denoted as,

7 $$\begin{aligned} {\begin{matrix} {S}_{q \leftrightarrow g} = \frac{{B}_q \cdot {B}_g}{\Vert {B}_q \Vert \Vert {B}_g \Vert } + \frac{{P}_q \cdot {P}_g}{\Vert {P}_q \Vert \Vert {P}_g \Vert } \end{matrix}} \end{aligned}$$

where *B* is the output feature in the Eq. (5) and *P* represents that the feature is extracted by the pre-trained model.

**Table 1:** Performance of different backbones before and after applying various methods on *COCO*.

Backbone	Method	mAP						*R*@*K*	AUROC
		All	Known	Unknown	Large	Medium	Small	All	All
MoCo^57^	Baseline	26.05	24.04	31.61	34.01	23.35	17.80	28.09	81.77
	FT	$29.78_{{\textbf {+3.73}}}$	$35.78_{{\textbf {+11.74}}}$	$13.16_{\underline{-18.45}}$	$34.31_{{\textbf {+0.30}}}$	$28.21_{{\textbf {+4.86}}}$	$25.13_{{\textbf {+7.33}}}$	$30.39_{{\textbf {+2.30}}}$	$74.03_{\underline{-7.74}}$
	TFA^51^	$28.89_{{\textbf {+2.84}}}$	$27.77_{{\textbf {+3.73}}}$	$31.99_{{\textbf {+0.38}}}$	$37.06_{{\textbf {+3.05}}}$	$26.24_{{\textbf {+2.89}}}$	$20.27_{{\textbf {+2.47}}}$	$30.78_{{\textbf {+2.69}}}$	$83.32_{{\textbf {+1.55}}}$
	Ours	$33.73_{{\textbf {+7.68}}}$	$34.02_{{\textbf {+9.98}}}$	$32.92_{{\textbf {+1.31}}}$	$42.75_{{\textbf {+8.74}}}$	$31.25_{{\textbf {+7.90}}}$	$23.65_{{\textbf {+5.85}}}$	$35.22_{{\textbf {+7.13}}}$	$86.21_{{\textbf {+4.44}}}$
RN50^58^	Baseline	29.36	27.37	34.88	36.74	27.63	20.75	30.84	82.73
	FT	$27.37_{\underline{-1.99}}$	$30.32_{{\textbf {+2.95}}}$	$19.20_{\underline{-15.68}}$	$34.96_{\underline{-1.78}}$	$24.38_{\underline{-3.25}}$	$20.04_{\underline{-0.71}}$	$28.80_{\underline{-2.04}}$	$81.79_{\underline{-0.94}}$
	TFA^51^	$31.68_{{\textbf {+2.32}}}$	$30.50_{{\textbf {+3.13}}}$	$34.96_{{\textbf {+0.08}}}$	$39.22_{{\textbf {+2.48}}}$	$29.92_{{\textbf {+2.29}}}$	$22.87_{{\textbf {+2.12}}}$	$33.04_{{\textbf {+2.20}}}$	$83.96_{{\textbf {+1.23}}}$
	Ours	$34.55_{{\textbf {+5.19}}}$	$33.92_{{\textbf {+6.55}}}$	$36.30_{{\textbf {+1.42}}}$	$42.65_{{\textbf {+5.91}}}$	$32.93_{{\textbf {+5.03}}}$	$24.72_{{\textbf {+3.97}}}$	$35.54_{{\textbf {+4.70}}}$	$86.02_{{\textbf {+3.29}}}$
Relabel^59^	Baseline	34.44	32.67	39.36	41.79	33.53	24.84	35.37	86.14
	FT	$30.32_{\underline{-4.12}}$	$33.41_{{\textbf {+0.74}}}$	$21.73_{\underline{-17.63}}$	$34.80_{\underline{-6.99}}$	$29.62_{\underline{-3.91}}$	$24.62_{\underline{-0.22}}$	$31.85_{\underline{-3.52}}$	$86.61_{{\textbf {+0.47}}}$
	TFA^51^	$36.96_{{\textbf {+2.52}}}$	$36.26_{{\textbf {+3.59}}}$	$38.90_{\underline{-0.46}}$	$44.46_{{\textbf {+2.67}}}$	$35.86_{{\textbf {+2.33}}}$	$27.37_{{\textbf {+2.53}}}$	$37.72_{{\textbf {+2.35}}}$	$86.63_{{\textbf {+0.49}}}$
	Ours	$40.75_{{\textbf {+6.31}}}$	$41.15_{{\textbf {+8.48}}}$	$39.65_{{\textbf {+0.29}}}$	$49.06_{{\textbf {+7.27}}}$	$39.72_{{\textbf {+6.19}}}$	$29.87_{{\textbf {+5.30}}}$	$40.96_{{\textbf {+5.59}}}$	$88.46_{{\textbf {+2.32}}}$
ViT-B^60^	Baseline	32.15	30.76	36.00	39.79	30.56	22.96	33.36	84.29
	FT	$32.61_{{\textbf {+0.46}}}$	$39.19_{{\textbf {+8.43}}}$	$14.36_{\underline{-21.64}}$	$35.65_{\underline{-4.14}}$	$31.71_{{\textbf {+1.15}}}$	$29.28_{{\textbf {+6.32}}}$	$33.44_{{\textbf {+0.08}}}$	$69.95_{\underline{-14.34}}$
	TFA^51^	$34.73_{{\textbf {+2.58}}}$	$34.86_{{\textbf {+4.10}}}$	$34.36_{\underline{-1.64}}$	$42.68_{{\textbf {+2.89}}}$	$32.88_{{\textbf {+2.32}}}$	$25.41_{{\textbf {+2.45}}}$	$35.94_{{\textbf {+2.58}}}$	$83.59_{\underline{-0.70}}$
	Ours	$37.81_{{\textbf {+5.66}}}$	$38.21_{{\textbf {+7.45}}}$	$36.73_{{\textbf {+0.73}}}$	$45.72_{{\textbf {+5.93}}}$	$36.14_{{\textbf {+5.58}}}$	$28.34_{{\textbf {+5.38}}}$	$38.38_{{\textbf {+5.02}}}$	$86.52_{{\textbf {+2.23}}}$
DeiT-B^61^	Baseline	35.94	35.26	37.82	44.85	34.72	24.42	37.91	79.21
	FT	$37.84_{{\textbf {+1.90}}}$	$46.19_{{\textbf {+10.93}}}$	$14.71_{\underline{-23.11}}$	$40.54_{\underline{-4.31}}$	$36.92_{{\textbf {+2.20}}}$	$35.07_{{\textbf {+10.65}}}$	$39.20_{{\textbf {+1.29}}}$	$72.38_{\underline{-6.83}}$
	TFA^51^	$38.77_{{\textbf {+2.83}}}$	$40.46_{{\textbf {+5.20}}}$	$34.09_{\underline{-3.73}}$	$46.60_{{\textbf {+1.75}}}$	$37.56_{{\textbf {+2.84}}}$	$28.85_{{\textbf {+4.43}}}$	$40.40_{{\textbf {+2.49}}}$	$80.78_{{\textbf {+1.57}}}$
	Ours	$46.20_{{\textbf {+10.26}}}$	$48.73_{{\textbf {+13.47}}}$	$39.20_{{\textbf {+1.38}}}$	$54.04_{{\textbf {+9.19}}}$	$45.30_{{\textbf {+10.58}}}$	$35.85_{{\textbf {+11.43}}}$	$46.04_{{\textbf {+8.13}}}$	$88.41_{{\textbf {+9.20}}}$
Swin-B^62^	Baseline	46.18	45.80	47.25	55.53	44.80	34.25	46.49	85.75
	FT	$38.50_{\underline{-7.68}}$	$45.69_{\underline{-0.11}}$	$18.56_{\underline{-28.69}}$	$40.94_{\underline{-14.59}}$	$37.81_{\underline{-6.99}}$	$35.80_{{\textbf {+1.84}}}$	$39.20_{\underline{-7.29}}$	$72.28_{\underline{-13.47}}$
	TFA^51^	$47.48_{{\textbf {+1.30}}}$	$49.25_{{\textbf {+3.45}}}$	$42.59_{\underline{-4.66}}$	$55.26_{\underline{-0.27}}$	$46.15_{{\textbf {+1.35}}}$	$37.76_{{\textbf {+3.51}}}$	$47.89_{{\textbf {+1.40}}}$	$84.21_{\underline{-1.54}}$
	Ours	$52.17_{{\textbf {+5.99}}}$	$53.05_{{\textbf {+7.25}}}$	$49.73_{{\textbf {+2.48}}}$	$60.75_{{\textbf {+5.22}}}$	$51.18_{{\textbf {+6.38}}}$	$40.86_{{\textbf {+6.61}}}$	$50.90_{{\textbf {+4.41}}}$	$91.35_{{\textbf {+5.60}}}$

Bold number and underline number respectively represent the improvement and reduction of baseline performance by the transfer method. See appendix for more results of *R*@*K* and AUROC.

## Experiments

In this section, we first introduce the experimental setting in Section “Experimental settings” and then employ our approach with other existing baselines on multiple benchmarks in Section “Main results”. Finally, we provide comprehensive ablation studies in Section “Ablation studies”.

### Experimental settings

#### Extending benchmarks

*COCO*^63^ is the most widely-used object detection benchmark for common entities, containing 80 categories, 112k images and 1.2M instances with exhaustive annotations. In order to simulate the open nature in *GOSO*, the 60 categories disjoint with *VOC*^64^ are used as known classes while the remaining 20 classes are used as unknown classes. Moreover, according to the number of queries in each category of ${D}^{Q}_{te}$, we further construct the small and large test set, in which there are 20 and 100 queries in each class correspondingly. *LVIS*^65^ is a more challenging benchmark for large-vocabulary instance segmentation, containing over 1200 entity labels and naturally retaining a long-tail distribution. As most of them contain few samples (even zero), we select the most frequent 400 categories and randomly split them into 300 for known classes and 100 for unknown, named *LVIS-400*. Compared to *COCO*, apart from more categories, *LVIS-400* contains more small instances ($< 32 \times 32$) and more semantic-similar classes, which greatly increase the difficulty of *GOSO*.

#### Evaluation protocol

To better demonstrate the performance of our model, we adopt three classic metrics into our evaluation: (1) mAP, the mean Average Precision, (2) *R*@*K*, Recall@Top-*K*, where *K* is the number of positive samples for the target query in the gallery, and (3) AUROC, the Area Under the Receiver Operating Characteristic curve, which is used to evaluate discrimination of retrieval score distribution between positive samples and negative samples in the gallery. For each of the above metrics, we report all results from six dimensions, that is, (1) All (all classes), Known (only known classes), Unknown (only unknown classes), Large (object area $\geqslant 96\times 96$), Medium ($32\times 32 <$ object area $< 96\times 96$) and Small (object area $\leqslant 32\times 32$). This allows us to observe trends in performance on various openness settings, as well as the impact of the object scale on our approach. Note that all results in the paper are obtained on the large test set (about 100 queries of each category) of each benchmark for reliability.

#### Implementation details

We train all models for 10 epochs using the AdamW optimizer^66^ on 8 GPUs with a batch size of 64. We set the initial learning rate as $\frac{batch size}{1024} \times 0.001$ and decay the learning rate to $1e^{-5}$ using the cosine schedule. We use a linear warm-up learning rate in the first epoch and apply gradient clipping to stabilize the training process. Moreover, to adapt the CNN structure, the feature maps of each layer in CNN are obtained after the *GAP* to $7\times 7$, and then flattened in the spatial dimension.

**Table 2:** Performance of different backbones before and after applying various methods on *LVIS-400*.

Backbone	Method	mAP						*R*@*K*	AUROC
		All	Known	Unknown	Large	Medium	Small	All	All
MoCo^57^	Baseline	9.93	10.63	7.90	14.39	8.71	6.81	12.46	78.96
	FT	$10.27_{{\textbf {+0.34}}}$	$11.74_{{\textbf {+1.11}}}$	$5.96_{\underline{-1.94}}$	$14.84_{{\textbf {+0.45}}}$	$8.75_{{\textbf {+0.04}}}$	$7.64_{{\textbf {+0.83}}}$	$12.33_{\underline{-0.13}}$	$75.00_{\underline{-3.96}}$
	TFA^51^	$10.89_{{\textbf {+0.96}}}$	$11.74_{{\textbf {+1.11}}}$	$8.41_{{\textbf {+0.51}}}$	$15.63_{{\textbf {+1.24}}}$	$9.59_{{\textbf {+0.88}}}$	$7.59_{{\textbf {+0.78}}}$	$13.47_{{\textbf {+1.01}}}$	$80.33_{{\textbf {+1.37}}}$
	Ours	$12.16_{{\textbf {+2.23}}}$	$13.20_{{\textbf {+2.57}}}$	$9.12_{{\textbf {+1.22}}}$	$17.59_{{\textbf {+3.20}}}$	$10.71_{{\textbf {+2.00}}}$	$8.29_{{\textbf {+1.48}}}$	$14.74_{{\textbf {+2.28}}}$	$81.54_{{\textbf {+2.58}}}$
RN50^58^	Baseline	11.20	11.85	9.30	15.98	9.91	7.81	12.46	78.96
	FT	$11.21_{{\textbf {+0.01}}}$	$12.72_{{\textbf {+0.87}}}$	$6.79_{\underline{-2.51}}$	$16.18_{{\textbf {+0.20}}}$	$9.74_{\underline{-0.17}}$	$7.96_{{\textbf {+0.15}}}$	$12.33_{\underline{-0.13}}$	$75.00_{\underline{-3.96}}$
	TFA^51^	$11.92_{{\textbf {+0.72}}}$	$12.68_{{\textbf {+0.83}}}$	$9.70_{{\textbf {+0.40}}}$	$16.92_{{\textbf {+0.94}}}$	$10.56_{{\textbf {+0.65}}}$	$8.39_{{\textbf {+0.58}}}$	$13.47_{{\textbf {+1.01}}}$	$80.33_{{\textbf {+1.37}}}$
	Ours	$12.85_{{\textbf {+1.65}}}$	$13.74_{{\textbf {+1.89}}}$	$10.24_{{\textbf {+0.94}}}$	$18.15_{{\textbf {+2.17}}}$	$11.44_{{\textbf {+1.53}}}$	$9.05_{{\textbf {+1.24}}}$	$14.74_{{\textbf {+2.28}}}$	$81.54_{{\textbf {+2.58}}}$
Relabel^59^	Baseline	13.71	14.50	11.40	18.36	12.51	10.28	16.38	82.7
	FT	$9.66_{\underline{-4.05}}$	$10.82_{\underline{-3.68}}$	$6.29_{\underline{-5.11}}$	$13.25_{\underline{-5.11}}$	$8.71_{\underline{-3.80}}$	$7.09_{\underline{-3.19}}$	$11.83_{\underline{-4.55}}$	$82.45_{\underline{-0.25}}$
	TFA^51^	$14.37_{{\textbf {+0.66}}}$	$15.30_{{\textbf {+0.80}}}$	$11.66_{{\textbf {+0.26}}}$	$19.33_{{\textbf {+0.97}}}$	$13.07_{{\textbf {+0.56}}}$	$10.77_{{\textbf {+0.49}}}$	$17.06_{{\textbf {+0.68}}}$	$82.91_{{\textbf {+0.21}}}$
	Ours	$15.18_{{\textbf {+1.47}}}$	$16.25_{{\textbf {+1.75}}}$	$12.06_{{\textbf {+0.66}}}$	$20.65_{{\textbf {+2.29}}}$	$13.79_{{\textbf {+1.28}}}$	$11.12_{{\textbf {+0.84}}}$	$17.78_{{\textbf {+1.40}}}$	$83.91_{{\textbf {+1.21}}}$
ViT-B^60^	Baseline	12.61	13.25	10.73	17.48	11.30	9.15	15.29	80.90
	FT	$8.74_{\underline{-3.87}}$	$10.50_{\underline{-2.75}}$	$3.62_{\underline{-7.11}}$	$12.43_{\underline{-5.05}}$	$7.59_{\underline{-3.71}}$	$6.47_{\underline{-2.68}}$	$11.01_{\underline{-4.28}}$	$66.71_{\underline{-14.19}}$
	TFA^51^	$13.98_{{\textbf {+1.37}}}$	$14.89_{{\textbf {+1.64}}}$	$11.33_{{\textbf {+0.60}}}$	$19.13_{{\textbf {+1.65}}}$	$12.62_{{\textbf {+1.32}}}$	$10.28_{{\textbf {+1.13}}}$	$16.79_{{\textbf {+1.50}}}$	$81.46_{{\textbf {+0.56}}}$
	Ours	$14.90_{{\textbf {+2.29}}}$	$15.89_{{\textbf {+2.64}}}$	$12.01_{{\textbf {+1.28}}}$	$20.36_{{\textbf {+2.88}}}$	$13.47_{{\textbf {+2.17}}}$	$10.93_{{\textbf {+1.78}}}$	$17.51_{{\textbf {+2.22}}}$	$82.60_{{\textbf {+1.70}}}$
DeiT-B^61^	Baseline	14.72	15.58	12.23	20.56	13.20	10.49	18.24	74.37
	FT	$10.23_{\underline{-4.49}}$	$12.44_{\underline{-3.14}}$	$3.80_{\underline{-8.43}}$	$14.21_{\underline{-6.35}}$	$9.04_{\underline{-4.16}}$	$7.64_{\underline{-2.85}}$	$12.88_{\underline{-5.36}}$	$66.88_{\underline{-7.49}}$
	TFA^51^	$16.03_{{\textbf {+1.31}}}$	$17.29_{{\textbf {+1.71}}}$	$12.39_{{\textbf {+0.16}}}$	$21.87_{{\textbf {+1.31}}}$	$14.51_{{\textbf {+1.31}}}$	$11.82_{{\textbf {+1.33}}}$	$19.52_{{\textbf {+1.28}}}$	$78.01_{{\textbf {+3.64}}}$
	Ours	$18.25_{{\textbf {+3.53}}}$	$19.71_{{\textbf {+4.13}}}$	$14.01_{{\textbf {+1.78}}}$	$24.53_{{\textbf {+3.97}}}$	$16.64_{{\textbf {+3.44}}}$	$13.67_{{\textbf {+3.18}}}$	$21.16_{{\textbf {+2.92}}}$	$83.51_{{\textbf {+9.14}}}$
Swin-B^62^	Baseline	19.39	20.19	17.04	25.82	17.77	14.60	22.49	80.61
	FT	$10.58_{\underline{-8.81}}$	$12.75_{\underline{-7.44}}$	$4.28_{\underline{-12.76}}$	$13.84_{\underline{-11.98}}$	$9.65_{\underline{-8.12}}$	$8.39_{\underline{-6.21}}$	$13.46_{\underline{-9.03}}$	$69.72_{\underline{-10.89}}$
	TFA^51^	$20.17_{{\textbf {+0.78}}}$	$21.37_{{\textbf {+1.18}}}$	$16.66_{\underline{-0.38}}$	$26.30_{{\textbf {+0.48}}}$	$18.60_{{\textbf {+0.83}}}$	$15.66_{{\textbf {+1.06}}}$	$23.38_{{\textbf {+0.89}}}$	$81.97_{{\textbf {+1.36}}}$
	Ours	$20.91_{{\textbf {+1.52}}}$	$22.06_{{\textbf {+1.87}}}$	$17.56_{{\textbf {+0.52}}}$	$27.33_{{\textbf {+1.51}}}$	$19.30_{{\textbf {+1.53}}}$	$16.13_{{\textbf {+1.53}}}$	$23.50_{{\textbf {+1.01}}}$	$86.43_{{\textbf {+5.82}}}$

Bold number and underline number respectively represent the improvement and reduction of baseline performance by the transfer method. See appendix for more results of *R*@*K* and AUROC.

### Main results

#### Comparisons with different training methods

All results are presented in Tables 1 and 2. First, we evaluate different training methods for *GOSO* including contrastive unsupervised learning (MoCo^57^), supervised learning, multi-label learning (Relabel^59^) based on ResNet-50 (RN50)^58^ trained on ImageNet. We find that the RN50 trained on Re-labeling ImageNet clearly outperforms other methods. Specifically, RN50-Relabel outperforms RN50 with supervised learning by almost 5%. In contrast, the performance of contrastive unsupervised training is much worse. This also proves that finer-grained annotation for multi-label can effectively improve the performance of *GOSO*.

#### Comparisons with different backbones

We compare different transformer-style architectures for *GOSO* including vision transformers (ViT^60^, DeiT^61^ and Swin Transformer^62^) trained on ImageNet in Tables 1 and 2. Compared to the RN50, ViT and DeiT achieve better performance on mAP and *R*@*K*, but do not show obvious advantages on AUROC. The local information is very important for the matching of multi-scale queries and galleries with multi-objects in *GOSO*. However, the structure of CNN is limited by inductive bias^67^, resulting in bias error. In contrast, Swin-Transformer gains almost 10% performance improvement. The hierarchical feature representation and multi-scale shifted windows in Swin-Transformer effectively promote the ability to extract effective local features for different scales objects in the gallery.

#### Comparisons with two finetuning methods

We compare our approach with two fine-tuning methods (*Fine-Tuning* and *Two-stage Fine-Tuning Approach*^51^). The evaluation protocols for these methods are detailed as follows:

- *Fine-Tuning* (FT) initializes the model with ImageNet pre-trained weights and subsequently fine-tunes it using multi-label classification on the provided known classes.
- *Two-stage Fine-Tuning Approach* (TFA)^51^ augments the frozen pre-trained model with an adaptive layer (e.g., a self-attention layer) appended after the last layer. This adaptive layer is then trained via multi-label classification using the known dataset. For evaluation, the proposed *OSF* is integrated into the TFA framework.

**Fig. 4:**
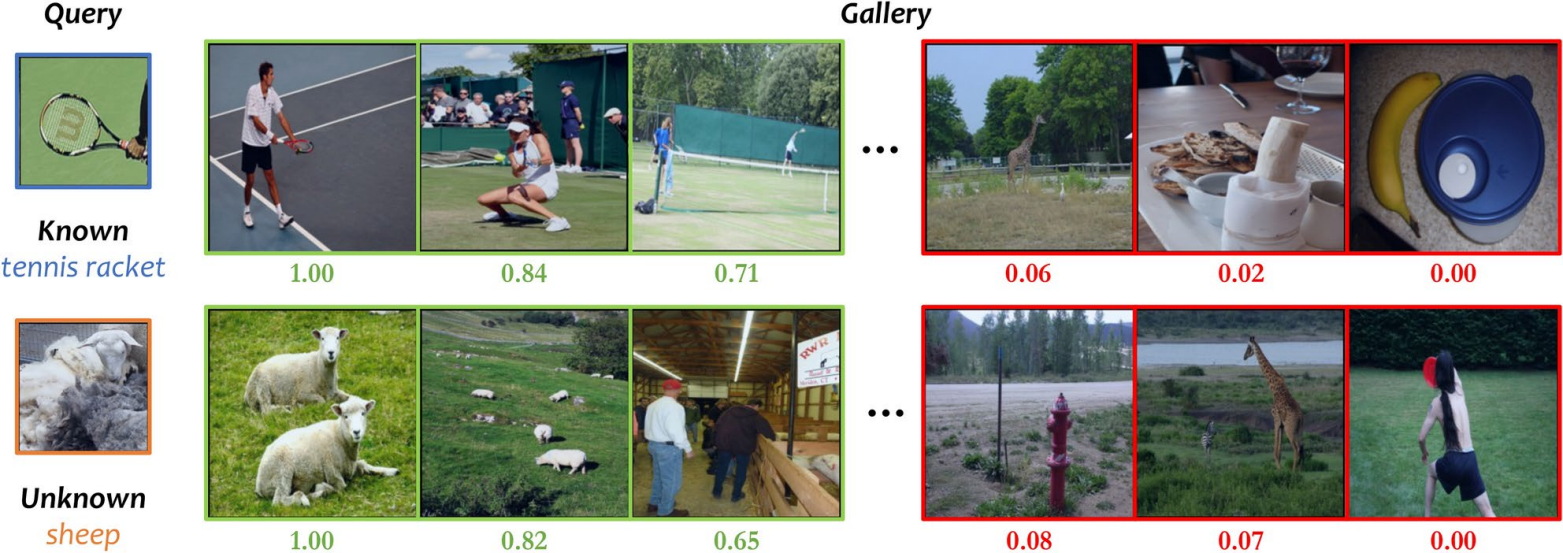
The retrieval results of our approach on known and unknown classes of *COCO*. Our method can effectively retrieve the corresponding images with small targets and multiple distractors.

As shown in Tables 1 and 2, our method clearly outperforms both of these approaches as well as the baseline. Notably, for the Deit-B backbone on the *COCO* dataset, our method achieves a significant improvement of 10% in mAP-All compared to the baseline. A closer analysis of the transfer methods relative to the baseline yields several reliable insights. While FT generally enhances performance on known classes across most backbones, it also tends to overfit these classes, which in turn degrades performance on unknown classes and adversely affects overall results. Meanwhile, we observed that while FT can sometimes boost overall performance, it occasionally leads to a decrease on known classes. We attribute this to the fact that our model is trained with a multi-class classification loss, which is not specifically designed for image matching tasks. Consequently, the learned feature representations may lack the necessary discriminative power and robustness for effective matching, as reflected in the mAP results. On the other hand, TFA with *OSF* mitigates the performance drop on unknown classes; however, much of this gain can be attributed directly to the proposed *OSF*, as evidenced in Table 5. Particularly with transformer-style backbones, TFA still suffers from overfitting on known classes, leading to declines in unknown class performance. In contrast, our method consistently boosts performance on both known and unknown categories across all backbones. This improvement is not solely due to the integration of *OSF*; it also stems from our novel query and gallery matching training mechanism based on *SEA-Net*. Moreover, our approach, which incorporates hierarchical feature fusion, promotes a more balanced improvement across large, medium, and small queries. Figure 4 illustrates several successful examples achieved by our method.

### Ablation studies

In this section, we first compare our *SEA* layer with different fusion schemes, then analyze the effects of our intra- and inter-stage scale adaptation. we finally show the importance of *OSF* to unknown object search in Open Reality.

**Table 3:** Ablation study with different fusion schemes on *COCO*.

Method	mAP			*R*@*K*		
	All	Known	Unknown	All	Known	Unknown
*AAF*	51.31	52.35	48.44	50.20	51.10	47.69
*SA*	51.68	52.86	48.41	50.42	51.54	47.34
*SEA*	**52.17**	**53.05**	**49.73**	**50.90**	**51.67**	**48.76**

All models are based on Swin-B.

#### Siamese exchanged attention layer

Table 3 shows the performance of different fusions schemes, including *AAF* (All-Attention Fusion), *SA* (Siamese-Attention does not exchange the attention map from two branches compared with *SEA*), *SEA* (Siamese Exchanged Attention). Among all these strategies, the proposed *SEA* achieves the best known, unknown, and overall performance on *COCO*. Despite the use of additional self-attention to combine information between two branches, all-attention with quadratic computation and memory complexity fails to achieve better performance. *SA* can be regarded as exchanging queries in two branches independent of self-attention, which reduces the consumption of computing and memory and achieves better performance than *AAT*. The *SEA* with exchanging the attention map fuses more useful information of the two branches than *SA*, so as to retain the common features more effectively.

**Table 4:** Ablation study with different combination of layers on *COCO*.

Layers	mAP				*R*@*K*		
	All	Large	Medium	Small	Large	Medium	Small
12	45.30	53.57	44.33	34.43	52.15	44.37	35.86
10–-12	45.92	**54.07**	44.93	35.23	52.72	44.97	36.73
7–12	46.04	53.98	45.12	35.55	52.72	45.16	37.01
4–12	46.04	54.01	45.09	35.58	**52.74**	45.14	37.04
1–4	45.80	53.45	44.12	34.63	52.09	44.56	36.03
5–8	45.83	53.51	44.01	34.52	52.00	44.43	36.00
9–12	46.01	54.05	45.03	35.31	52.63	45.00	37.05
1–12	**46.20**	54.04	**45.30**	**35.85**	52.69	**45.31**	**37.23**

Significant values are in bold.

All models are based on DeiT-B with 12 layers.

#### Inter-stage scale adaptation

We perform experiments to understand the effect of inter-stage scale adaptation by testing the different layers combinations in Table 4. We keep the last layer containing more semantics and gradually add front layers. It can be observed that the combination of the last layer and more front layers achieves higher overall performance. Specifically, when only the last several layers are used, better performance is achieved on the large objects search. With the addition of shallow features, *SEA-Net* has better performance for small and medium objects. On the whole, the combination of all layer features has the best generalization in the search of objects with different scales.

#### Open score fusion module

Although TFA could maintain unknown performance on several backbones, its gains come from the better generalization ability of the pre-training model. Furthermore, both our method and TFA have different degrees of over-fitting on the known classes, which degrades the performance of the unknown classes as shown in Table 5. Without the help of the pre-training retrieval score, our method not only achieves the best performance on known classes but also performs better than TFA on unknown classes. In addition, the performance of *SEA* on known classes slightly exceeds the performance of *SEA*+*OSF*. Nevertheless, the *OSF* is still more suitable for open reality with unknown categories.

#### Computational complexity analysis

To further evaluate the efficiency of our proposed approach, we analyze the computational complexity in terms of both FLOPs and the number of trainable parameters. Specifically, before incorporating our module, our model requires approximately 15.19 GMac of FLOPs and contains 86.83 million parameters. After integrating our module, the computational cost increases to 36.15 GMac, with the total parameter count rising to 118.92 million. Although the inclusion of our module introduces additional computational overhead, the significant performance improvements observed in our experiments justify this trade-off. This analysis demonstrates that our method achieves a favorable balance between computational efficiency and enhanced matching performance in open reality settings.

**Table 5:** Ablation study for *OSF* on *COCO* with DeiT-B backbone.

Method	mAP			*R*@*K*		
	All	Known	Unknown	All	Known	Unknown
Baseline	35.94	35.26	37.82	37.91	37.40	39.30
TFA	34.48	37.73	25.49	35.80	39.09	26.69
TFA+*OSF*	**38.77**	**40.46**	**34.09**	**40.40**	**42.20**	**35.40**
SEA	44.91	**49.73**	31.54	44.93	**49.34**	32.70
SEA+*OSF*	**46.20**	48.73	**39.20**	**46.04**	48.43	**39.43**

Significant values are in bold.

## Discussion and conclusion

This paper extends a valuable and practical vision task, *GOSO*. Based on multiple granularity stage-stacked fusion mechanisms, we propose an effective matching architecture, that is, *SEA-Net*. Then, an open score fusion module is adapted during inference for more robust matching scores for both known and unknown queries. Our method achieves SOTA performance on two *GOSO* evaluation benchmarks. In the future, how to make full use of known classes to improve the generalization of the model on unknown classes is worth further exploration, including but not limited to questing the impact of the number and range of known classes.

## Supplementary Information

Supplementary Information.

## Data Availability

The datasets generated during and/or analyzed during the current study are available from the corresponding author upon reasonable request.
