# Supplementary material for: Toward general object search in open reality
Source: Sci Rep. 2025 Apr 19;15:13523. doi: 10.1038/s41598-025-97251-5 (PMC12009333; doi:10.1038/s41598-025-97251-5)
Supplement: Supplementary file 1 — Supplementary Information. [file 41598_2025_97251_MOESM1_ESM.pdf]

Supplementary Material

In this supplementary material, we provide additional details which we could not include in the main paper due to space limitations, including more discussions and experimental analysis that help us develop further insights to the *GOSO* task and our proposed approach. We discuss:

- Why the proposed *GOSO* is a valuable task?
- Additional details of extending benchmarks.
- A brief introduction of compared methods.
- More thorough results of $R@K$ and AUROC.
- Do we need an ImageNet pre-trained model?

A Why *GOSO* is a Valuable Task?

One of the primary goals of computer vision is image understanding, which involves numerous tasks including recognizing ***what*** objects are present (classification), localizing ***where*** are the objects (detection) and characterizing ***which*** relationships (same or different) between multiple objects (retrieval). However, the newly extending *GOSO* focuses more on determining ***whether*** a query object exists in gallery images or not (searching), which is demanded to *decouple the composition of actual everyday scenes through matching with a much lower model complexity*.

#1: Find better solutions for object search. To this end, the most straightforward way is adopting sliding windows over multiple scales paradigm on images or feature maps. However, it is difficult to unify the density and scale of the windows, which leads to the inefficiency and impracticality of this scheme. Another feasible solution is the detection pipeline, which has been extensively studied in recent years. But considering that object search only focuses on the problem of whether containing or not, it does not need precise location information, three disadvantages of detection arise: 1) The localization branch requires redundant annotations and increases the complexity of searching. 2) Expensive labeling costs reduce the practicality. 3) Inductive bias leads to the inability to open reality. We hope that *GOSO* defines the problem of object search more appropriately and attracts further research into this open topic.

#2: Be Beneficial for other visual tasks. In fact, the problems that *GOSO* solve are also encountered in some other existing visual tasks, *e.g.*,, single object tracking (SOT), image caption and weakly-supervised detection, which roughly involve a similar process of object searching. It is worth mentioning that *GOSO* goes one step further with extending the problem space to general objects in open reality. We believe that researches on *GOSO* can not only improve the scene understanding ability of deep models but also boost the performance upper bound of these existing tasks. In addition, there are many industrial vision applications that can be enhanced. We explain this below.

#3: A Pre-Filter for Detection Applications. Consider a detection task in the actual scenario: there are M pictures in a large-scale database, where N images contain the specified target (denoted as X) representing user needs, where $N << M$. The purpose of one deep model is to find these N pictures and annotate the locations (*e.g.*,, bounding box) of X. Assume that the computational complexity of a detection operation is O_d and a matching operation is O_m. In order to complete the above task, obviously, the amount of computation for the conventional detection pipeline is MO_d. However, another feasible pipeline is first utilizing the *GOSO* model to perform object searching on these M pictures and then employing the detection pipeline on the N^+ potentially candidate images obtained by *GOSO* model, where $N \leqslant N^+ << M$. And the amount of computation for the second pipeline is $MO_m + N^+O_d$. Then the computation reduction Δ can be further expressed as:

$$\begin{aligned} \Delta &= MO_d - (MO_m + N^+O_d) \\ &= M \times (O_d - O_m) - N^+O_d \end{aligned} \tag{8}$$

Based on the essential assumption of *GOSO* that $O_m < O_d$, then $\Delta > 0$ can be approximately obtained when $N^+ << M$. And the greater of the M or the smaller of the N^+, the more computation reduction of the detection system.

#4: An Auxiliary Module for Auto-annotator. With the continuous accumulation of massive amounts of data, how to purposefully reduce the candidate images space is a key part of the automatic labeling pipeline. Since the manual annotation is time-consuming and labor-intensive, *GOSO* provides an efficient alternative way for shrinking the data space automatically in the open scenario. Furthermore, the *GOSO* can deal with general objects, not limited to known classes, which is indispensable for auto-labeling systems.

B More Details of Extending Benchmarks

B.1 COCO

In order to simulate the open nature of *GOSO*, we first choose the 60 categories disjoint with *VOC*[61] as known classes while the remaining 20 classes as unknown. The *COCO* are composed of two sets, *i.e.*,, training set and test set. For a concrete instantiation of object searching, the training set is then naturally designed to contain *gallery set* and *query set*, which only include known classes. Among them, the gallery set is directly transferred from the training set of *COCO (detection)*, while the query set is obtained by cropping instances from the gallery set without omission. And the test set has roughly the same construction logic, while the only difference is that the test set contains all categories, and about 100 (or about 20) query images are provided for each category in the large (or small) version. Note that our experimental results are obtained from the large test set for the stability and robustness. We summarize the statistical information of *COCO* in the Table 6 and show its scale distribution for different query sets in the Fig.5.

Table 6. The proposed *COCO* evaluation benchmark for *GOSO*.

	Train Set		Test Set (small)		Test Set (large)	
	Query	Gallery	Query	Gallery	Query	Gallery
# classes	60	60	80	80	80	80
# images	980461	98459	1580	5000	7543	5000
# img/cls	16341	1641	19.8	62.5	94.3	62.5

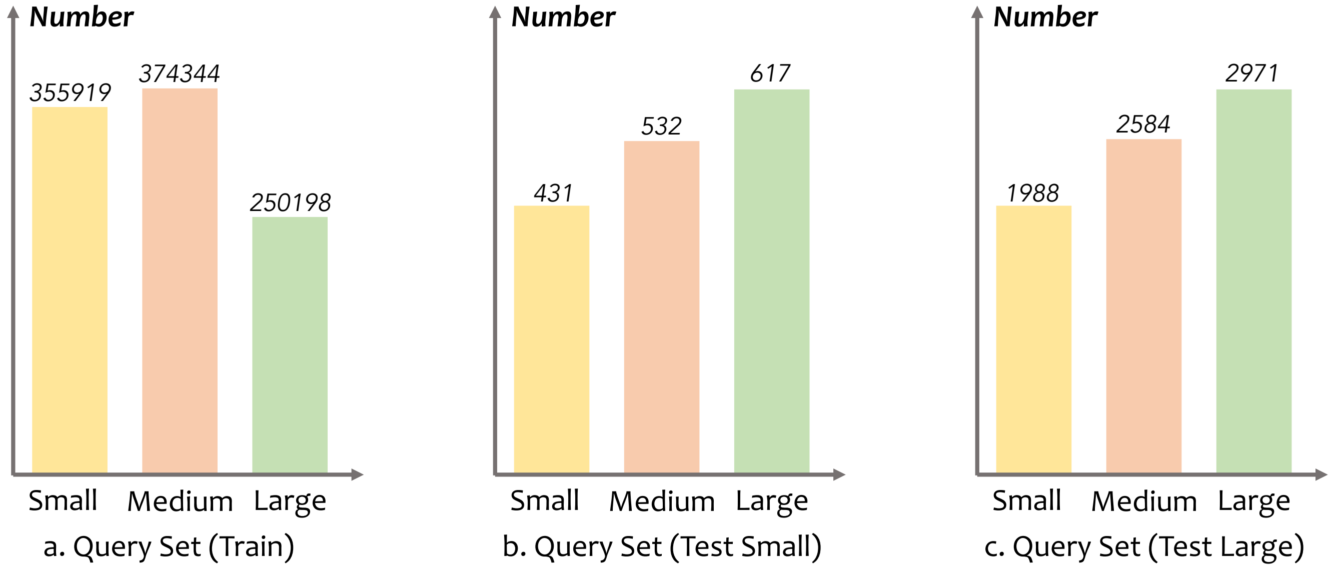

Figure 5. The scale distribution of different query sets on *COCO*. The small/medium/large are $s < 32^2, 32^2 \leqslant s \leqslant 96^2, s > 96^2$, where s is the area of query.

B.2 LVIS-400

For the sake of making the *GOSO* evaluation closer to the category distribution in reality, we further extend *LVIS*[62] as a more difficult benchamrk. Following the filter rule of containing at least 20 instances, we select 400 categories from *LVIS*, *i.e.*,, *LVIS-400*, to form the total category space, which is about $5\times$ that of *COCO*. Then 300 categories are randomly selected as known classes, and the remaining 100 categories are regarded as unknown. The rest of the construction process is similar to *COCO*. And we summarize the statistical number of *LVIS-400* in the Table 7 and show its scale distribution for different query sets in the Fig.6.

C A Brief Introduction of Compared Methods

Three Training Methods for RN50. As mentioned in the main paper, we compare three training paradigms based on ResNet-50 (RN50)[55] and supplement their details below:

Table 7. The proposed *LVIS-400* evaluation benchmark for *GOSO*.

	Train Set		Test Set (small)		Test Set (large)	
	Query	Gallery	Query	Gallery	Query	Gallery
# classes	300	300	400	400	400	400
# images	907306	89496	8000	19809	35826	19809
# img/cls	3024.4	298.3	20	49.5	89.6	49.5

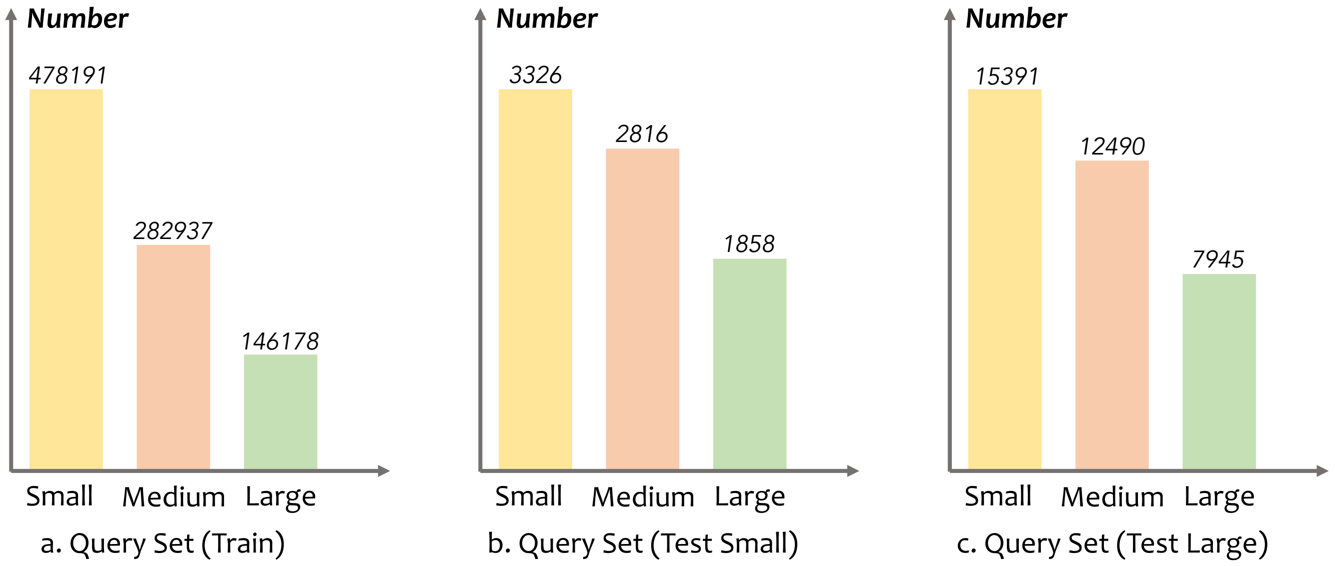

Figure 6. The scale distribution of different query sets on *LVIS-400*. Note the small/medium/large is consist with the Fig.5

- **MoCo**[54] is a way of building large and consistent dictionaries for unsupervised learning with a contrastive loss. The pre-trained MoCov2[65] is used in our paper with top-1 accuracy of 71.1% on ImageNet.
- **RN50**[55] indicates normal supervised classification training on ImageNet. The pre-trained RN50 model has 80.4% top-1 accuracy on ImageNet.
- **Relabel**[56] represents RN50 trained by multi-label classification on ImageNet with exhaustive multi-label annotations per image[56]. The pre-trained Relabel model attains the top-1 classification accuracy of 80.2% with the CutMix regularization on ImageNet.

Three Transformer-Style Architectures. Moreover, we supplement the details of three transformer-based vision backbones mentioned in the main paper as follows:

- **ViT**[57] is a pioneering work with directly applying a Transformer architecture on non-overlapping medium-sized image patches for image classification. The pre-trained ViT-B/32[57] with large-scale training datasets has 80.7% top-1 classification accuracy on ImageNet.
- **DeiT**[58] introduces several training strategies that allow ViT to be effective with using smaller ImageNet dataset. The pre-trained DeiT-B[58] is used in our paper with top-1 accuracy of 82.0% on ImageNet.
- **Swin Transformer**[59] is a hierarchical Transformer whose representation is computed with shifted windows, which could be as several local self-attention. The pre-trained Swin-B attains the top-1 classification accuracy of 85.3% on ImageNet.

D More Results

More thorough results of $R@K$ and AUROC are provided in Table 8 and Table 9. It can be seen that the performance trend of $R@K$ and AUROC is basically consistent with that of mAP in the main paper. First, exhaustive multi-label annotations can effectively improve the performance of *GOSO*. Second, the hierarchical local self-attentions in Swin-Transformer greatly promote the ability to extract effective features for objects of different scales in the gallery. Finally, our approach with *SEA* module and *OSF* layer achieves more improvements on known, unknown, large, medium, and small queries.

Table 8. $R@K$ and AUROC of different backbones before and after applying various methods on *COCO*. Red and Blue respectively represent the improvement and reduction of baseline performance by the transfer method.

Backbone	Method	$R@K$					AUROC				
		Known	Unknown	Large	Medium	Small	Known	Unknown	Large	Medium	Small
MoCo[54]	Baseline	26.26	33.17	35.33	25.65	20.58	82.05	81.02	85.12	81.32	77.45
	FT	$36.21_{+9.95}$	$14.26_{-18.91}$	$34.66_{-0.67}$	$28.85_{+3.20}$	$26.10_{+5.52}$	$77.14_{-4.91}$	$65.42_{-15.60}$	$77.32_{-7.80}$	$72.26_{-9.06}$	$71.47_{-5.98}$
	TFA[48]	$29.81_{+3.55}$	$33.47_{+0.30}$	$38.01_{+2.68}$	$28.49_{+2.84}$	$23.09_{+2.51}$	$84.16_{+2.11}$	$81.00_{-0.02}$	$86.54_{+1.42}$	$82.69_{+1.37}$	$79.41_{+1.96}$
	Ours	$35.52_{+9.26}$	$34.38_{+1.21}$	$43.05_{+7.72}$	$33.18_{+7.53}$	$26.34_{+5.76}$	$87.75_{+5.70}$	$81.95_{+0.93}$	$88.88_{+3.76}$	$85.82_{+4.50}$	$82.80_{+5.35}$
RN50[55]	Baseline	29.13	35.6	37.29	29.38	23.25	83.12	81.65	85.15	82.9	78.98
	FT	$31.62_{+2.49}$	$20.99_{-14.61}$	$35.89_{-1.40}$	$26.11_{-3.27}$	$21.82_{-1.43}$	$84.00_{+0.88}$	$75.69_{-5.96}$	$85.83_{+0.68}$	$80.61_{-2.29}$	$77.39_{-1.59}$
	TFA[48]	$32.11_{+2.98}$	$35.64_{+0.04}$	$39.60_{+2.31}$	$31.52_{+2.14}$	$25.36_{+2.11}$	$84.81_{+1.69}$	$81.06_{-0.05}$	$86.22_{+1.07}$	$83.97_{+1.07}$	$80.64_{+1.66}$
	Ours	$35.06_{+5.93}$	$36.88_{+1.28}$	$42.55_{+5.26}$	$34.16_{+4.78}$	$27.04_{+3.79}$	$87.13_{+4.01}$	$82.93_{+1.28}$	$88.24_{+3.09}$	$86.10_{+3.20}$	$82.66_{+3.68}$
Relabel[56]	Baseline	33.81	39.69	42.11	34.50	26.59	86.73	84.52	88.96	86.16	81.98
	FT	$34.66_{+0.85}$	$24.09_{-15.60}$	$35.91_{-6.20}$	$31.34_{-3.16}$	$26.56_{-0.03}$	$88.90_{+2.17}$	$80.29_{-4.23}$	$88.17_{-0.79}$	$86.52_{+0.36}$	$84.47_{+2.49}$
	TFA[48]	$37.19_{+3.38}$	$39.20_{-0.49}$	$44.47_{+2.36}$	$36.73_{+2.23}$	$29.07_{+2.48}$	$87.79_{+1.06}$	$83.41_{-1.11}$	$89.35_{+0.39}$	$86.36_{+0.20}$	$82.97_{+0.99}$
	Ours	$41.36_{+7.55}$	$39.85_{+0.16}$	$48.29_{+6.18}$	$40.08_{+5.58}$	$31.33_{+4.74}$	$89.95_{+3.22}$	$84.35_{-0.17}$	$90.96_{+2.00}$	$88.34_{+2.18}$	$84.97_{+2.99}$
ViT-B[57]	Baseline	32.09	36.89	40.07	32.04	25.2	84.91	82.58	87.38	84.13	79.99
	FT	$39.66_{+7.57}$	$16.20_{-20.69}$	$36.50_{-3.57}$	$32.33_{+0.29}$	$30.35_{+5.15}$	$73.17_{-11.74}$	$61.00_{-21.58}$	$71.51_{-15.87}$	$68.98_{-15.15}$	$68.89_{-11.10}$
	TFA[48]	$36.15_{+4.06}$	$35.35_{-1.54}$	$42.87_{+2.80}$	$34.43_{+2.39}$	$27.68_{+2.48}$	$84.72_{-0.19}$	$80.44_{-2.14}$	$86.73_{-0.65}$	$82.98_{-1.15}$	$79.76_{-0.23}$
	Ours	$38.83_{+6.74}$	$37.11_{+0.22}$	$45.13_{+5.06}$	$37.07_{+5.03}$	$30.13_{+4.93}$	$87.67_{+2.76}$	$83.35_{+0.77}$	$89.26_{+1.88}$	$85.95_{+1.82}$	$83.24_{+3.25}$
DeiT-B[58]	Baseline	37.4	39.3	45.67	36.9	27.79	80.32	76.14	82.92	79.3	73.66
	FT	$47.24_{+9.84}$	$16.94_{-22.36}$	$41.73_{-3.94}$	$38.22_{+1.32}$	$36.75_{+8.96}$	$76.47_{-3.85}$	$61.05_{-15.09}$	$73.67_{-9.25}$	$71.49_{-7.81}$	$71.63_{-2.03}$
	TFA[48]	$42.20_{+4.80}$	$35.40_{-3.90}$	$47.28_{+1.61}$	$39.36_{+2.46}$	$31.63_{+3.84}$	$82.98_{+2.26}$	$74.69_{-1.45}$	$83.44_{+0.52}$	$80.29_{+0.99}$	$77.51_{+3.85}$
	Ours	$48.43_{+11.03}$	$39.43_{+0.13}$	$52.69_{+7.02}$	$45.31_{+8.41}$	$37.23_{+9.44}$	$90.51_{+10.19}$	$82.56_{+6.46}$	$90.33_{+7.41}$	$88.10_{+8.80}$	$86.00_{+12.34}$
Swin-B[59]	Baseline	46.22	47.22	54.69	45.24	36.03	87.05	82.15	88.25	85.53	82.37
	FT	$45.87_{-0.35}$	$20.74_{-26.48}$	$41.62_{-13.07}$	$38.40_{-6.84}$	$36.69_{+0.66}$	$75.35_{-11.70}$	$63.80_{-18.35}$	$73.96_{-14.29}$	$71.22_{-14.31}$	$71.18_{-11.19}$
	TFA[48]	$49.81_{+3.59}$	$42.60_{-4.62}$	$54.74_{+0.05}$	$46.77_{+1.53}$	$39.29_{+3.26}$	$86.25_{-0.80}$	$78.53_{-3.62}$	$86.24_{-2.01}$	$83.43_{-2.10}$	$82.22_{-0.15}$
	Ours	$51.67_{+5.45}$	$48.76_{+1.54}$	$58.37_{+3.68}$	$50.06_{+4.82}$	$41.02_{+4.99}$	$92.88_{+5.83}$	$87.12_{+4.97}$	$93.02_{+4.77}$	$91.31_{+5.78}$	$88.96_{+6.59}$

Table 9. $R@K$ and AUROC of different backbones before and after applying various methods on *LVIS-400*. Red and Blue respectively represent the improvement and reduction of baseline performance by the transfer method.

Backbone	Method	$R@K$					AUROC				
		Known	Unknown	Large	Medium	Small	Known	Unknown	Large	Medium	Small
MoCo[54]	Baseline	13.21	10.31	17.51	11.07	8.98	79.42	77.61	82.22	78.52	75.71
	FT	$14.01_{+0.80}$	$7.47_{-2.84}$	$17.26_{-0.25}$	$10.69_{-0.38}$	$9.54_{+0.56}$	$76.50_{-2.92}$	$70.63_{-6.98}$	$79.06_{-3.16}$	$73.91_{-4.61}$	$72.12_{-3.59}$
	TFA[48]	$14.35_{+1.14}$	$10.89_{+0.58}$	$18.65_{+1.14}$	$12.03_{+0.96}$	$9.89_{+0.91}$	$81.00_{+1.58}$	$78.38_{+0.77}$	$83.49_{+1.27}$	$79.84_{+1.32}$	$77.32_{+1.61}$
	Ours	$15.79_{+2.58}$	$11.67_{+1.36}$	$20.56_{+3.05}$	$13.17_{+2.10}$	$10.62_{+1.64}$	$82.34_{+2.92}$	$79.23_{+1.62}$	$84.89_{+2.67}$	$81.03_{+2.51}$	$78.34_{+2.63}$
RN50[55]	Baseline	14.79	12.28	19.34	12.77	10.42	79.54	78.39	82.09	78.93	76.26
	FT	$15.10_{+0.31}$	$8.48_{-3.80}$	$18.79_{-0.55}$	$11.84_{-0.93}$	$9.83_{-0.59}$	$83.01_{+3.47}$	$78.35_{-0.04}$	$85.34_{+3.25}$	$81.10_{+2.17}$	$78.85_{+2.59}$
	TFA[48]	$15.64_{+0.85}$	$12.69_{+0.41}$	$20.23_{+0.89}$	$13.45_{+0.68}$	$11.09_{+0.67}$	$80.75_{+1.21}$	$78.98_{+0.59}$	$83.11_{+1.02}$	$79.93_{+1.00}$	$77.47_{+1.21}$
	Ours	$16.61_{+1.82}$	$13.20_{+0.92}$	$21.33_{+1.99}$	$14.28_{+1.51}$	$11.71_{+1.29}$	$82.36_{+2.82}$	$80.24_{+1.85}$	$84.62_{+2.53}$	$81.44_{+2.51}$	$79.04_{+2.78}$
Relabel[56]	Baseline	17.12	14.2	21.48	15.09	12.57	83.15	81.41	85.48	82.27	80.07
	FT	$13.16_{-3.96}$	$7.97_{-6.23}$	$15.78_{-5.70}$	$10.80_{-4.29}$	$8.97_{-3.60}$	$83.51_{+0.36}$	$79.38_{-2.03}$	$84.97_{-0.51}$	$81.97_{-0.30}$	$80.27_{+0.20}$
	TFA[48]	$17.95_{+0.83}$	$14.48_{+0.28}$	$22.38_{+0.90}$	$15.70_{+0.61}$	$13.15_{+0.58}$	$83.58_{+0.43}$	$80.99_{-0.42}$	$85.77_{+0.29}$	$82.41_{+0.14}$	$80.34_{+0.27}$
	Ours	$18.79_{+1.67}$	$14.85_{+0.65}$	$23.56_{+2.08}$	$16.34_{+1.25}$	$13.46_{+0.89}$	$84.59_{+1.44}$	$81.90_{+0.49}$	$86.94_{+1.46}$	$83.35_{+1.08}$	$81.21_{+1.14}$
ViT-B[57]	Baseline	15.89	13.54	20.39	13.95	11.62	81.33	79.66	83.83	80.42	78.20
	FT	$13.02_{-2.87}$	$5.14_{-8.40}$	$15.32_{-5.07}$	$9.67_{-4.28}$	$8.33_{-3.29}$	$68.86_{-12.47}$	$60.45_{-19.21}$	$69.80_{-14.03}$	$66.01_{-14.41}$	$64.26_{-13.94}$
	TFA[48]	$17.67_{+1.78}$	$14.26_{+0.72}$	$22.06_{+1.67}$	$15.42_{+1.47}$	$12.97_{+1.35}$	$82.19_{+0.86}$	$79.35_{-0.31}$	$84.48_{+0.65}$	$80.94_{+0.52}$	$78.71_{+0.51}$
	Ours	$18.46_{+2.57}$	$14.75_{+1.21}$	$23.11_{+2.72}$	$16.07_{+2.12}$	$13.40_{+1.78}$	$83.38_{+2.05}$	$80.31_{+0.65}$	$85.90_{+2.07}$	$82.01_{+1.59}$	$79.61_{+1.41}$
DeiT-B[58]	Baseline	19.06	15.87	24.15	16.71	13.94	74.89	72.84	78.44	73.91	70.13
	FT	$15.42_{-3.64}$	$5.51_{-10.36}$	$17.50_{-6.65}$	$11.49_{-5.22}$	$9.94_{-4.00}$	$69.43_{-5.46}$	$59.44_{-13.40}$	$69.71_{-8.73}$	$66.14_{-7.77}$	$64.80_{-5.33}$
	TFA[48]	$20.76_{+1.70}$	$15.94_{+0.07}$	$25.27_{+1.12}$	$18.05_{+1.34}$	$15.03_{+1.36}$	$78.98_{+4.09}$	$75.20_{+2.36}$	$81.34_{+2.90}$	$77.49_{+3.58}$	$74.88_{+4.75}$
	Ours	$22.54_{+3.48}$	$17.15_{+1.28}$	$27.23_{+3.08}$	$19.60_{+2.89}$	$16.71_{+2.77}$	$84.57_{+9.68}$	$80.42_{+7.58}$	$86.55_{+8.11}$	$83.04_{+9.13}$	$80.60_{+10.47}$
Swin-B[59]	Baseline	23.24	20.30	28.70	20.90	17.92	81.11	79.15	83.80	80.04	77.74
	FT	$15.87_{-7.37}$	$6.44_{-13.86}$	$17.12_{-11.58}$	$12.42_{-8.48}$	$10.98_{-6.94}$	$72.17_{-8.94}$	$62.58_{-16.57}$	$72.27_{-11.53}$	$69.18_{-10.86}$	$67.60_{-10.14}$
	TFA[48]	$24.52_{+1.28}$	$20.05_{-0.25}$	$29.28_{+0.58}$	$21.86_{+0.96}$	$19.06_{+1.14}$	$83.05_{+1.94}$	$78.82_{-0.33}$	$84.95_{+1.15}$	$81.35_{+1.31}$	$79.46_{+1.72}$
	Ours	$24.59_{+1.35}$	$20.34_{+0.04}$	$29.61_{+0.91}$	$21.95_{+1.05}$	$18.98_{+1.06}$	$87.16_{+6.05}$	$84.31_{+5.16}$	$89.34_{+5.54}$	$85.95_{+5.91}$	$83.74_{+6.00}$

E Do we need ImageNet pre-trained models?

In order to further verify the superiority of our method, we introduce a stricter setting for *GOSO*: *NO ImageNet Pre-train* model, which academically meets the open set setting more rigorously, *i.e.*, the unknown is indeed not seen by the model unintentional. To be concrete, we first train the multi-label classification model on all known classes (60 classes on *COCO* or 300 on *LVIS-400*), and then replace the ImageNet pre-trained model with this converged backbone. Note the remaining part is consistent with the description in our main paper for a fair comparison. We conduct experiments on two benchmarks with Transformer-based models, and all results are summarized in Table 10. It can be seen that our proposed framework still

Table 10. Performance of different backbones trained from scratch (*NO ImageNet Pretrain*) before and after applying our method. Red represent the improvement of baseline performance by the transfer method.

Dataset	Backbone	Method	mAP						$R@K$	AUROC
			All	Known	Unknown	Large	Medium	Small	All	All
COCO	ViT-B[57]	Baseline	15.80	17.17	12.00	16.85	15.41	14.75	18.75	68.70
		Ours	16.66+0.86	18.05+0.88	12.84+0.84	17.71+0.86	16.26+0.85	15.65+0.90	19.61+0.86	71.24+2.54
	DeiT-B[58]	Baseline	19.84	22.56	12.3	21.55	19.37	17.93	22.59	70.34
		Ours	20.77+0.93	23.75+1.19	12.54+0.24	22.39+0.84	20.32+0.95	18.99+1.06	23.61+1.02	71.36+1.02
	Swin-B[59]	Baseline	21.99	24.80	14.20	24.58	20.96	19.50	24.70	73.88
		Ours	23.98+1.99	27.22+2.42	15.01+0.81	26.60+2.02	23.12+2.16	21.24+1.74	26.68+1.98	76.23+2.35
LVIS	ViT-B[57]	Baseline	4.70	5.19	3.27	6.09	4.22	3.94	6.35	64.16
		Ours	4.90+0.20	5.43+0.24	3.38+0.11	6.30+0.21	4.43+0.21	4.13+0.19	6.57+0.22	65.19+1.03
	DeiT-B[58]	Baseline	5.72	6.42	3.69	7.72	5.04	4.62	7.74	64.66
		Ours	5.92+0.20	6.66+0.24	3.77+0.08	7.92+0.20	5.26+0.22	4.78+0.16	8.00+0.26	64.95+0.29
	Swin-B[59]	Baseline	7.04	7.95	4.39	9.52	6.22	5.62	9.08	70.40
		Ours	7.59+0.55	8.61+0.66	4.63+0.24	10.08+0.56	6.79+0.57	6.13+0.51	9.77+0.69	71.96+1.56

consistently maintains a good performance improvement, which demonstrates the effectiveness of the approach. Although this stricter setting can effectively prevent the leakage of unknown categories, we have also obtained the following conclusions through experiments: 1) The performance is generally severely degraded before and after. Obviously, this is caused by a serious decline in feature generalization ability. One path to solving this problem is to be paved by pre-training a feature representation that is as universal as possible. 2) Multi-label classification pre-training on known space without a good initialization converges very slowly. According to the claims of[66], the pre-training can speed up the convergence processes of downstream tasks. In summary, we believe that *ImageNet Pretrain* is not necessary for *GOSO*, but it is extremely beneficial, especially in the actual scenarios with the nature of ever-changing and open-ended.
